# Movement Disorders and Oculomotor Abnormalities in Whipple’s Disease: An Updated Systematic Review

**DOI:** 10.5334/tohm.1075

**Published:** 2025-11-11

**Authors:** Errikos Maslias, Ruben Anker, Philip Euskirchen, Karin Diserens, Julien F. Bally

**Affiliations:** 1 Service of Neurology, Department of Clinical Neurosciences, Lausanne University Hospital (CHUV) and University of Lausanne, Lausanne, Switzerland; 2 University of Lausanne, Lausanne, Switzerland; 3 German Cancer Consortium (DKTK), Partner Site Berlin, German Cancer Research Center (DKFZ), Heidelberg, Germany; 4 Department of Neuropathology, Charité-Universitätsmedizin Berlin, corporate member of Freie Universität Berlin and Humboldt Universität zu Berlin, Berlin, Germany

**Keywords:** Whipple’s disease, central nervous system, movement disorders, oculomotor abnormalities, systematic review

## Abstract

**Introduction:**

Whipple’s disease (WhD) is a rare multisystemic infection caused by *Tropheryma whipplei*, with central nervous system (CNS) involvement seen in up to 50% of cases. Neurological symptoms may precede systemic features or occur in isolation. Movement disorders (MDs) and oculomotor abnormalities, especially oculomasticatory myorhythmia (OMM) and oculofacioskeletal myorhythmia (OFSM), are of a high diagnostic importance but remain underrecognized. This systematic review aims to update our understanding of MDs in CNS-WhD, building on a 2018 review.

**Methods:**

A systematic search of MEDLINE, EMBASE, and Cochrane Library was performed for English-language human studies published between 01/2017-05/2025. Search terms targeted WhD and MDs. Titles, abstracts and full-text were screened in Rayyan.ai, by two independent reviewers.

**Results:**

We added 19 articles (22 new cases) to the 100 articles (146 cases) from the previous report, making up a total of 168 CNS-WhD patients with MDs or oculomotor abnormalities. Supranuclear gaze palsy (SGP) was the most common sign (58%), followed by myorhythmia and ataxia (40% each). Pathognomonic OMM/OFSM were identified in 25% of cases, higher than previously reported. MRI showed abnormalities in 87% of cases, and brain tissue PAS staining had the highest diagnostic yield, although mostly performed post-mortem. Treatment with ceftriaxone followed by Trimethoprim-Sulfamethoxazole remained common, though doxycycline–hydroxychloroquine use has increased. MDs improved in 53% of cases.

**Conclusion:**

Oculomotor abnormalities and MDs, especially SGP and OMM/OFSM/other myorhythmia, are key diagnostic clues in CNS-WhD, even in the absence of systemic symptoms. Greater diagnostic awareness is essential to improve outcomes of this life-threatening, but treatable, condition.

## Introduction

Whipple’s disease (WhD) is a rare multisystemic infectious disorder caused by the actinobacterium *Tropheryma whipplei* with an estimated incidence of 0.4 to 1.0 per 1,000,000 a year [1,2,3]. The gastrointestinal system is most commonly affected, followed by the central nervous system, joints and other organs. The classical presentation of WhD is diarrhea, abdominal pain, arthralgia and weight loss. Other symptoms include low-grade fever, anaemia, and adenopathy [4]. The reported prevalence of clinical CNS involvement varies widely, ranging from 6% to 63%, with most estimates falling between 10% and 43% [1,2,5,6,7,8,9,10,11,12,13,14]. Notably, post-mortem studies reveal central nervous system (CNS) involvement in up to 50% of cases, most frequently in a periventricular and periaqueductal distribution [15,16,17,18,19]. In approximately 5% of cases, the initial manifestations are exclusively neurological [6,11,20,21,22]. Although neurological involvement was once considered a late manifestation of Whipple’s disease, it is now often the first clinical sign and poses the greatest risk for long-term disability [23].

Central nervous system Whipple’s disease (CNS-WhD) can mimic almost any neurological disorder [24]; its most common neurological manifestations include cognitive changes, encephalopathy, neuropsychiatric alterations (such as depression, personality changes and apathy), increased appetite, sleep disturbances, oculomotor abnormalities and movement disorders (MDs) [11,21,22,25,26,27], either accompanying systemic symptoms of classical WhD or, in rarer cases, as isolated neurological presentations [28]. The MDs include diverse symptoms and signs, ranging from the relatively rare pathognomonic ones like oculo-masticatory myorhythmia (OMM) and oculofacioskeletal myorhythmia (OFSM), to less specific but more frequent signs, such as vertical supranuclear gaze palsy (SGP), ataxia, and, less frequently, parkinsonism and other MDs.

The diagnosis remains challenging due to variable clinical presentations and the necessity of integrating histological, molecular, and imaging findings. The hallmark of CNS-WhD is OMM and OFSM, which are pathognomonic. However, in its absence, a combination of neurological symptoms along with either positive periodic acid-Schiff (PAS) staining of macrophages (mostly performed in the lamina propria of duodenal biopsies) or a positive polymerase chain reaction (PCR) analysis for *Tropheryma whipplei* (performed in various body fluids like the saliva or blood) is required for definitive diagnosis. CNS involvement can also be confirmed through PCR testing of cerebrospinal fluid (CSF) or brain biopsy, in which case PAS staining is also performed; but sensitivity and specificity concerns persist, whatever the technique or body fluid/sample. MRI findings frequently demonstrate mediobasal and periventricular involvement, but a normal MRI does not exclude CNS-WhD.

The treatment of CNS-WhD remains a subject of debate, with no consensus on the optimal therapeutic approach. While traditional regimens such as intravenous ceftriaxone followed by oral trimethoprim-sulfamethoxazole (TS) have been widely used, concerns about relapse [29] and treatment failure have led to alternative strategies including combinations of doxycycline and hydroxychloroquine [28,29,30]. These controversial findings highlight the complexity of treating CNS-WhD. Given the high mortality and relapse rates, rapid diagnosis and optimal treatment remain critical.

The data on neurological manifestations of WhD remain scarce, limited to case reports and small case series. This invited systematic review aims to update knowledge on the prevalence, clinical manifestations, and diagnostic significance of MDs in CNS-WhD, building on a previous 2018 review and incorporating findings from recent case studies and case series. This review highlights the importance of early recognition of CNS-WhD related MDs, with the goal of improving diagnostic accuracy, allowing for a rapid initiation of antibiotic therapy to improve patient outcomes.

## Methods

### Search Method and Eligibility Criteria

A comprehensive search strategy was developed using medical subject headings (MeSH) and keywords related to WhD and MDs. The search was based on the original strategies from Bally et al., adapted by a scientific librarian documentalist of the medical library of Lausanne. Thesaurus terms were verified for each platform to optimize retrieval. The search was restricted to studies published from 01.01.2017, onward (postdating the last original search in Bally et al.). Alerts were created on each platform to update the search periodically until 01.05.2025. Searches were conducted in EMBASE.com, MEDLINE OVID, and COCHRANELIBRARY.com, limited to human-subject studies and English-language. The two first co-authors (EM and RA) independently screened all titles and abstracts using the RAYYAN.ai software, employing a blinded approach to maintain objectivity in study selection. Any disagreements between the reviewers were resolved by a movement disorder specialist (JFB).

For articles identified as potentially eligible, full-text versions were assessed for final inclusion. Cases in which MDs are secondary to systemic WhD, but not related to direct parenchymal CNS involvement, were excluded. No formal quality appraisal tool was applied to these case reports, given the descriptive nature of the data. Instead, we included only reports with adequate clinical detail, as guided by the Population, Exposure, and Outcome (PEO) criteria. We acknowledge that the heterogeneity and potential biases inherent in the case reports are limitations of this analysis, and results should be interpreted with caution. Where available, published video footage of the patients’ examinations was reviewed to verify the described movement disorder phenomenology (among the 22 new cases, video material was available and examined for 2 cases; one further case had a video that the authors could not access).

**Figure 1:**
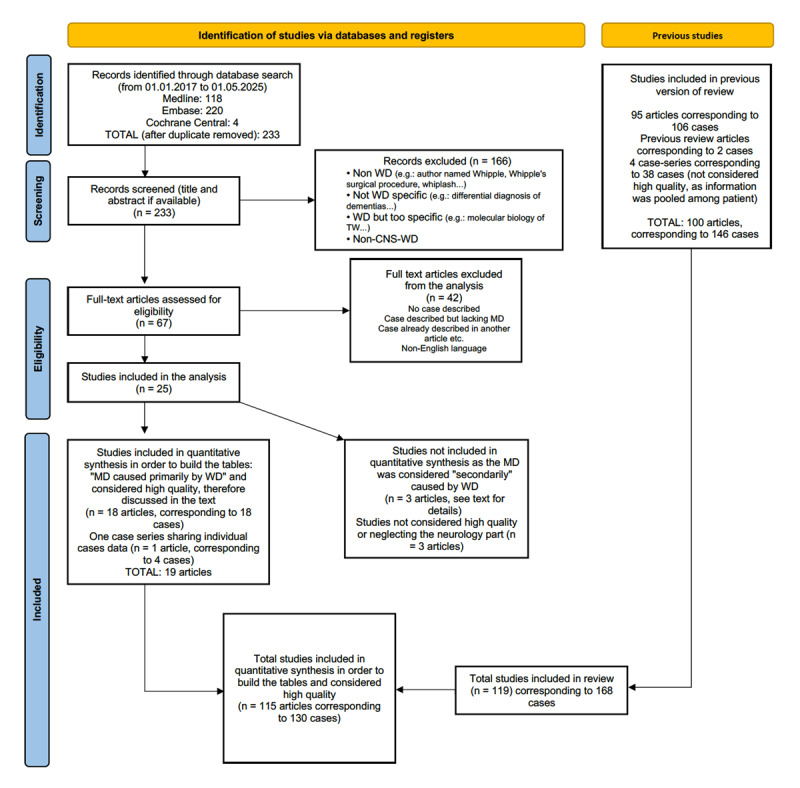
PRISMA flowchart of the systematic review update.

Abstracts and full-text articles were stored and deduplicated using the Zotero software. A PRISMA flowchart was created to document the selection process (Figure 1).

### Risk of Bias in Individual Studies and Data Collection Process

To minimize potential biases, a comprehensive search strategy was implemented to ensure broad coverage of CNS-WhD cases. The review was further restricted to cases with sufficient clinical details. However, the limitations of incomplete may still pose challenges (i.e. the lack of description for a particular sign cannot confirm its absence). Data extraction was conducted using Excel, where information was extracted from each eligible study. Two reviewers split the studies equally, each performing a single extraction for their assigned articles.

### Data Synthesis

Descriptive statistics were conducted using frequency tables to assess MDs, ocular motility disorders, laboratory findings, imaging and EEG findings, and treatment outcomes. When data on treatment or outcomes were unavailable, they were excluded from percentage calculations.

### Definitions of Clinical Signs and Symptoms

The definition and classification of the various clinical signs were made consistent with the 2018 review by Bally et al. [31] to ensure continuity and standardization. These definitions, available in the original article, are re-pasted in the supplemental material.

## Results

In total, the present article sums up 119 articles (19 new and 100 from the previous review – which spanned 99 articles and itself reported two new cases, making it the 100^th^ article), of which only four were case series, comprising a total of 168 patients with CNS-WhD and at least one movement disorder or typical oculomotor abnormalities. Between 01.01.2017 and 01.05.2025, which corresponds to the update period of this systematic review, we collected and analysed data from 22 cases, 18 coming from single case reports, and four coming from a case series that shared individual case data [32]. These were then combined with the data from 146 cases previously presented in the 2018 review by Bally et al. (Figure 1). Among the 168 patients identified, only 130 were sufficiently well-described to allow for inclusion in the quantitative analysis; all percentages reported below are based on these 130 cases. Of the 168 patients, 78% were male and 22% were female, with a mean age of onset of 49.8 years and the median duration of symptoms before diagnosis was approximately 2 years.

### Movement Disorders and Oculomotor Abnormalities

Supranuclear gaze palsy (SGP) emerged as the most prevalent clinical sign, reported in 75 individuals (58%), with vertical gaze most commonly affected initially (Table 1B). Among the 62 patients (48%) exhibiting vertical SGP, later progression to horizontal gaze restriction occurred in 36 cases (58%), while 26 (42%) remained limited to vertical impairment. In most instances, SGP was not isolated but associated with other MDs (62 of 75 patients, 83%). Only 13 out of 75 (17%) individuals exhibited SGP in the absence of a recognized movement disorder, and within this subgroup, cognitive impairment was the most frequent additional finding, occurring in all but one case with isolated SGP.

Among MDs (Table 1A), myorhythmia and ataxia were the most frequently documented, each affecting 40% of the 130 well-described patients (n = 52). Within this group with myorhythmia, 32 patients (25% of the 130 well-documented cases) displayed the pathognomonic oculomasticatory or oculofacioskeletal myorhythmia (OMM/OFSM). Among these 32, when oculomotor features were well detailed (n = 23), pendular nystagmus of vergence type was noted in 19 patients (83%), while vertical nystagmus was described in 4 cases (17%). Palatal movements were mentioned in only 2 patients (6%), and persistence of the OMM/OFSM during sleep was noted in 9 cases (28%).

**Table 1A:** Movement disorders in CNS-WhD patients suffering from at least one MD or SGP.


	TOTAL (n = 168)	SINGLE CASE (n = 130)	SINGLE CASE % (IF TOTALNOT 130)	CASE SERIES (n = 38)	CASE SERIES %

Age at MD onset (if specified; otherwise at first CNS sign onset or first hospital consultation), years	49.4	49.8	–	48.3	–

Men	129	102	**78**	27	**71**

Women	39	28	**22**	11	**29**

**Myoclonus** (including myorhythmia): total	77	69a	**53**	8	**21**

**I. Myorhythmia**: total^a^	57	52b	**40**	5	**13**

1. OMM and OFSM: rhythmic pendular convergent-divergent or vertical nystagmus associated with masticatory or facioskeletal myorhythmia	36	32	25	4	11

a. Vergence component mentioned	9	19	**83 (23)**	NA	NA

b. Vertical component mentioned	4	4	**17 (23)**	NA	NA

c. Involving palate at the same frequency	2	2	6 (32)	NA	NA

d. Persistence in sleep mentioned	9	9	**28 (32)**	NA	NA

2. Isolated ocular myorhythmia not associated with masticatory or facioskeletal myorhythmia	4	4	3	0	0

3. Myorhythmia (either rhythmic, continuous, or the word myorhythmia was used by the authors) of branchial/spinal muscles without any ocular involvement	17	17	13	0	0

a. Branchial muscles	6	6	**35 (17)**	NA	NA

b. Spinal muscles	8	8	**47 (17)**	NA	NA

c. Both branchial and spinal muscles	3	3	**18 (17)**	NA	NA

**II. Arrhythmic myoclonus:** only nonrhythmic forms or not specified whether rhythmic or not^b^	21	18	14	3	8

a. Branchial muscles	2	2	12 (17)	NA	NA

b. Spinal muscles	5	5	**29 (17)**	NA	NA

c. Both branchial and spinal muscles or not specified	11	11	**65 (17)**	NA	NA

**Stimulus-sensitivity specified**	1	1	6 (17)	NA	NA

**Ataxia:** total	72	52	**40**	20	53

1. Appendicular ataxia: total/isolated	28	24|12	**18|9**	4/NA	11/NA

2. Axial ataxia: total/isolated	29	24|12	**18|9**	5/NA	13/NA

3. Both appendicular and axial ataxia	12	12	9	NA	NA

4. Unspecified ataxia	27	16	12	11	**29**

**Gait and balance abnormalities:** total	67	60	**46**	7	**18**

1. Gait problems (any type)	46	41	**32**	5	13

2. Postural/balance issues (any type) and/or falls	31	29	**22**	2	5

3. Both	10	10	8	NA	NA

**Other movement disorders** ranked in frequency					

1a. Parkinsonism (all possible cases)	25	17	13	8	**21**

1b. Parkinsonism (only unequivocal)	7	6	5	1	3

2. Tremor	13	10	8	3	8

3. Dystonia (including 4 with isolated blepharospasm)	8	7	5	1	3

4. Bruxism	3	3	2	0	0

5. Chorea	3	1	1	2	5


Except for the age row and the percentage columns, all numbers refer to number of cases. Percentages are highlighted in bold when the number of patients is large enough for percentages to be considered relevant. a. 32 + 4 + 17 = 53, but total of patients is 52 because one patient had first isolated ocular then isolated facial myorhythmia. b. There were 18 arrhythmic myoclonus + 52 myorhythmia = 70, but total number of patients was 69 because 1 patient had both arrhythmic myoclonus and myorhythmia. NA, not applicable because of pooling of information in the case series.

Additional oculomotor disturbances (Table 1B) were observed in 22% of patients (n = 29), predominantly involving diplopia or isolated cranial nerve palsies. Involuntary eye movements, including ocular myorhythmia and nystagmus, were reported in 48 patients (37%). Among them, the majority (n = 32, 25%) were classified as OMM or OFSM, while 4 cases (3%) exhibited purely ocular myorhythmia without associated masticatory or skeletal involvement. Other involuntary eye movements, mainly nystagmus, were reported in 12 patients (9%). Overall, ocular myorhythmias were primarily grouped within the OMM/OFSM phenotype.

**Table 1B:** Ocular motility disorders in CNS-WhD patients suffering from at least one MD or SGP.


	TOTAL (n = 168)	SINGLE CASE (n = 130)	SINGLE CASE % (IF TOTALNOT 130)	CASE SERIES (n = 38)	CASE SERIES %

**Ocular motility palsies/paresis**					

1. Total SGP	87	75	**58**	12	**32**

a. Associated with at least one MD	62	62	**83 (75)**	NA	NA

b. Not associated with any MD	13	13	**17 (75)**	NA	NA

2. Vertical SGP	65	62	**48**	3	8

a. Vertical alone	28	26	**42 (62)**	2	**67 (3)**

b. Associated with horizontal	37	36	**58 (62)**	1	**33 (3)**

3. SGP not specified whether vertical or horizontal	22	13	10	9	**24**

4. Isolated horizontal gaze palsy (not specified whether supranuclear)	2	2	2	0	0

5. Non–supranuclear palsies/paresis (isolated palsy of any of the 3 oculomotor nerves or mention of diplopia; blurred vision excluded, as it lacks precision regarding its cause)	29	29	**22**	0	0

**Spontaneous involuntary eye movements:** total	53	48	**37**	5	13

1. OMM and OFSM	See table 1A	See table 1A	–	See table 1A	–

2. Isolated ocular myorhythmia	See table 1A	See table 1A	–	See table 1A	–

3. Other involuntary eye movements (mostly nystagmus)	13	12	9	1	3


Except for the percentages’ columns, all numbers refer to number of cases. Percentages are highlighted in bold when the number of patients is large enough for percentages to be considered relevant. NA, not applicable because of pooling of information in the case series.

Non-rhythmic or unspecified myoclonus was reported in 18 cases (14%). Ataxia, either of appendicular, axial, or mixed type, was present in 52 patients (40%). Furthermore, 60 individuals (46%) experienced gait disturbances or balance deficits, which were frequently associated with underlying static and gait ataxia.

Other MDs occurred less frequently, each affecting fewer than 10% of cases. These included parkinsonism, dystonia, tremor, chorea, and bruxism, underscoring the heterogeneity of motor presentations in CNS-WhD. Such variability necessitates a high index of suspicion and careful neurological assessment to identify patterns suggestive of this rare but treatable condition.

### Other Neurological Signs

In addition to MDs, various other neurological signs were observed (Supplemental Table 1). Cognitive impairment was the most common, affecting 69% of patients. Encephalopathy occurred in 52%, while 42% displayed neuropsychiatric symptoms such as mood changes or apathy. Sleep disturbances were present in 40%.

Signs of corticospinal tract involvement, including spasticity and hyperreflexia, were reported in 31% of cases, and dysarthria in 29%. Hypothalamic dysfunction (e.g. eating disorders), seizures, and headaches were each seen in 16% of cases.

Other neurological features were less frequent, each affecting fewer than 10% of patients. These included in descending order of frequency ptosis, visual disturbances (blurred vision or reduced acuity), sensory deficits, and muscular weakness and some others.

### Systemic, Non-neurological Signs

Systemic involvement was frequently reported. Nearly half of the patients (48%) experienced weight loss, while fever occurred in 35% of cases. Arthritis was noted in 38%, and gastrointestinal complaints were present in 41%. Lymphadenopathy was documented in 18% of cases. In contrast, cardiac involvement and cutaneous hyperpigmentation were uncommon, each observed in approximately 5% of patients.

### Laboratory, Imaging, and Electroencephalographic Findings

Radiological abnormalities were identified on MRI in 87% of the 82 evaluated patients (Table 2). The predominant pattern consisted of multiple T2-weighted hyperintensities, reported in 77% of cases. Contrast enhancement was observed in nearly a quarter of patients (24%), occasionally involving mediobasal or periventricular regions. Signs of cerebral atrophy were less frequent, present in 15% of cases. EEG was abnormal in 57% of the 54 recordings available, with the majority showing nonspecific findings such as diffuse slowing (77%), and a minority displaying epileptiform discharges (23%).

**Table 2:** Laboratory, imaging, and electroencephalographic findings in CNS-WhD patients suffering from at least one MD or SGP.


	SINGLE CASE (n = 130)	POSITIVITY %	CASE SERIES (n = 38)	POSITIVITY%

**Laboratory/microscopy**				

CSF pleocytosis (>5 cells/mm^3^): present/total number reported	44|93	**47**	6|16	**38**

CSF protein elevation (>0,45 g/L): present/total number reported	36|89	**40**	7|16	**44**

CSF PAS: positive/total number reported	10|27	**37**	2|2	**100**

CSF PCR: positive/total number reported	34|48	**71**	11|12	**92**

Blood PCR: positive/total number reported	11|18	**61**	0|0	NA

Gut PAS: positive/total number reported	44|84	**52**	19a|28	**68**

Gut PCR: positive/total number reported	14|24	**58**	14|16	**88**

Lymph-node PAS: positive/total number reported	11|14	**79**	0|0	NA

Lymph node PCR: positive/total number reported	2|3	**67**	0|0	NA

Brain (biopsy or autopsy) PAS: positive/total number reported	37|39	**95**	9|9	**100**

Brain (biopsy or autopsy) PCR: positive/total number reported	4|6	**67**	0|0	NA

Electron microscopy (EM): positive/total number reported	24|29	**83**	1|1	**100**

1. Gut EM: positive (% over 24 — respectively 1 — cases)	12	**50**	1	**100**

2. Brain EM: positive (% over 24 cases)	11	**46**	0	0

3. CSF EM: positive (% over 24 cases)	1	4	0	0

4. Lymph nodes EM: positive (% over 24 cases)	2	8	0	0

**Brain imaging**				

**Brain-CT:** abnormal/total number reported	20|40	50	NA	NA

Type of abnormality				

1. Atrophy (% over 20 cases)	7	35	–	–

2. Lesion (hypodensity or mass-like) (% over 20 cases)	10	50	–	–

3. Enhancement (% over 20 cases)	5	25	–	–

**Brain MRI:** abnormal/total number reported	71|82	87	NA	NA

Type of abnormality				

1. Atrophy (%over 71 cases)	11	15	–	–

2. Lesion (either spontaneous T2 hyperintensity or mass-like lesion) (% over 71 cases)	55	77	–	–

3. Enhancement (%over 71 cases)	17	24	–	–

4. other/nonspecific (%over 71 cases)	8	11	–	–

**Electroencephalography (EEG)**				

EEG: abnormal/total number reported	31|54	57	NA	NA

1. Epileptiform discharges (% over 31 cases)	7	23	–	–

2. Non pecific abnormalities (% over 31 cases)	24	77	–	–


Except for the percentages’ columns, all numbers refer to number of patients. Percentages are highlighted in bold when the number of patients is large enough for percentages to be considered relevant. a: Imprecise number because of methodological issues of 1 case series: means “up to 19 positive PAS staining in the gut.” NA, not applicable.

Among diagnostic tools, duodenal biopsy was the most frequently employed. PAS staining was positive in 52% of the 84 cases where it was performed, while PCR confirmed *T. whipplei* DNA in 58% of the 24 biopsies tested.

CSF analysis showed abnormal parameters, such as pleocytosis or elevated protein in nearly 50% of cases; PAS positivity was noted in 37% of the 27 samples tested, while PCR achieved a higher sensitivity, with a 71% positivity rate (34 out of 48).

In cases where a brain tissue biopsy was obtained, the biopsy site was chosen based on MRI findings, i.e. targeted to an accessible lesion or area of abnormal signal. Brain tissue analysis (on biopsy or autopsy) had the highest diagnostic yield: PAS staining was positive in 95% (37/39), and PCR in 67% (4/6).

Electron microscopy on tissue samples, most often from brain or intestine, demonstrated typical bacterial structures in 83% of 29 cases. Blood PCR was positive in 61% of the 18 cases. Additional diagnostic sites included saliva, feces, lymph nodes, intraocular fluid [33], and others.

### Treatment and Outcome

The most frequently administered antibiotics were trimethoprim-sulfamethoxazole (TS), used in 70% of cases, and ceftriaxone, reported in 51% (Table 3). The combination of doxycycline and hydroxychloroquine, has shown a noticeable increase in recent years. It was administered in 8/22 (19%) newly added cases, compared to only 12/106 (14%) cases in the previous cohort.

**Table 3:** Treatment outcome and antibiotics used in 130 CNS-WhD suffering from at least one MD or SGP.


OUTCOME / ANTIBIOTICS	SINGLE CASES	%

**Outcome**	**n = 130**	

1. Improvement (from partial to good)	64	49

2. No improvement/worsening	13	10

3. Death	32	25

4. Not specifically mentioned if the clinical picture improved	21	16

**Pooling categories into good and bad outcomes**	**n = 109** ^a^	

1. good outcome	64	59

2. bad outcome (no improvement/worsening/death)	45	41

**Antibiotics**	**n = 103** ^b, c^	

1. Trimethoprim-sulfamethoxazole	72	70

2. Ceftriaxone	53	51

3. Penicillin (any type)	20	19

4. Chloramphenicol	16	16

5. Tetracycline	23	22

6. Streptomycin	13	13

7. Doxycycline (twice with hydroxychloroquine)	20	19


a. n = 109 because we excluded cases for whom outcome was not reported. b. No antibiotics used or not mentioned what antibiotics were used in n = 27; total cases = 103. c. Most frequent antibiotics are used in combination.

Treatment outcomes were reported in 109 patients. Among these, clinical improvement was observed in 59%, while 10% experienced a worsening of their condition, and 25% died. When focusing specifically on MDs, therapeutic response was reported in 53% of cases (58/109). However, the lack of detailed reporting often prevented precise correlation between antibiotic therapy and the evolution of specific neurological signs. In some instances, certain symptoms such as myorhythmia showed improvement while others, like supranuclear gaze palsy (SGP), remained unchanged, highlighting the heterogeneity of treatment response.

## Discussion

This updated systematic review provides an in-depth evaluation of the MDs and oculomotor abnormalities associated with CNS-WhD, through the analysis of 119 articles including 168 CNS-WhD patients. By integrating both historical data from the 2018 review by Bally et al. [31] and recent case reports, this study enhances the clinical understanding of this diagnostically challenging condition.

Both the rarity of the disease and the wide spectrum and frequent absence of specific clinical signs contribute to the diagnostic complexity. However, the identification of MDs that are pathognomonic for CNS-WhD, such as OMM and OFSM, or the presence of a combination of highly suggestive signs, including the classic triad of SGP, myoclonus of any kind (comprising myorhythmia, classified among the rhythmic myoclonus in the present review) and cognitive impairment [34,35], is essential, especially in cases where systemic symptoms are absent or minimal. Ataxia being equally prevalent as myorhythmia (40%), and gait disorders in general making up to 46% of cases, this triad could be augmented by these two signs. Recognizing a combination of these five signs can therefore facilitate timely diagnosis, enabling prompt initiation of appropriate treatment and potentially improving patient outcomes.

### Semiology

Supranuclear gaze palsy (SGP) was the most frequently reported oculomotor abnormality, found in approximately two thirds of the patients included, with initial vertical involvement in nearly half. This supports earlier reports [11,21,24] and emphasizes the role of detailed oculomotor examination in identifying CNS-WhD. Importantly, SGP frequently co-occurred with MDs, especially myorhythmia and ataxia, suggesting it is rarely isolated in CNS-WhD.

Myoclonus of any kind was the most prominent movement disorder [36], with myorhythmia being its most frequent subtype, occurring in 40% of cases in our review. OMM and OFSM, were described as early as 1963 [37] but was not reported to be specific to WhD until 1986 [38]. Nowadays, it is established that these movement disorders are pathognomonic of CNS-WhD [39]. Our review highlights their presence in one-quarter of patients, a prevalence notably higher than the 8% to 20% previously reported in the literature, and sensibly more frequent than in Bally et al. 2018 [11,25,31,40,41], which may be attributed to increased clinical awareness.

Ataxia emerged as another prevalent sign (40%), in line with previous estimates of 10–55% [21,42]. Ataxia in CNS-WhD disease may present as both axial and appendicular, often coexisting within the same patient. However, isolated ataxia is uncommon [31].

Other MDs, including parkinsonism, dystonia, chorea, and tremor, were each observed in <10%, reflecting their lower specificity. This clinical variability highlights the importance of considering CNS-WhD when facing persistent MDs of unknown origin.

Concerning non-oculomotor and non-MDs symptoms, sleep disturbances were the most prevalent, presenting in 40% of cases of CNS-WhD, a frequency consistent with previous reports [43,44,45]. Disruptions of the sleep-wake cycle have been described as part of the neurologic presentation of WhD, with manifestations ranging from excessive somnolence to transient total sleep loss [45,46]. These sleep disturbances are likely due to direct affection of diencephalic structures by the pathogen [45].

### Ophthalmic Whipple’s Disease

In the present update, 5/22 new cases presented with uveitis [33,47,48,49,50], one even as an unprecedented unique case description starting with orbital inflammatory syndrome at the onset of the WhD [50]. This high ratio made us re-analyse the raw data of the 2018 review, which yielded one additional case of uveitis, making up a total of 5% prevalence of this ophthalmologic condition.

Given these findings, ophthalmologists should consider WhD in the differential diagnosis of atypical or treatment-resistant uveitis, especially when accompanied by systemic or neurologic symptoms. The apparent rise in ocular involvement may reflect increased clinical awareness and systematic ophthalmologic assessments, but may also be influenced by a publication bias, favouring unusual ophthalmologically interesting cases.

### Diagnosis: Laboratory, Imaging, and Electroencephalography

Our findings confirm the diagnostic challenges inherent to CNS-WhD. Brain MRI abnormalities were present in 87% of cases, but nonspecific, necessitating histological or molecular confirmation. PAS performed on brain tissue showed the highest diagnostic yield (95%); the test was done either on brain biopsy or on post-mortem tissues, however, in vivo PAS staining may be less feasible due to the technical difficulty of obtaining adequate brain tissue samples. PAS staining on brain tissue was negative in only two cases, but with WhD diagnosis confirmation by PAS positivity on gut biopsy associated with CNS symptoms and signs.

Duodenal biopsy remained the most frequently performed test, but with an insufficient sensitivity of about 50–60% in our cohort, after PAS staining or tissue PCR. This is somewhat lower than the 72.6% frequency on PCR in duodenal biopsies reported in the literature [51,52]. This approach also suffers from low specificity, as PAS-positive macrophages are also found in other infectious and non-infectious conditions (i.e. leishmaniasis, endemic mycoses, and lysosomal storage disorders such as Niemann-Pick type B disease) [53]. Moreover, CNS-WhD, though frequently accompanied by digestive tract signs, also exists in its CNS isolated form, or without gastro-intestinal involvement; the gut may therefore not be the best tissue for confirming all CNS-WhD.

This technique also often necessitates multiple samples due to the focal and patchy distribution of the disease in the duodenal mucosa [4,52,54]. According to the literature, the detection of *T. whipplei’s* bacterial 16S RNA gene in stools and in saliva through PCR has a sensitivity of 57.7%–84% and a high specificity of 97.6% [51], although it is only anecdotally mentioned in our dataset. These high sensitivity and specificity percentages must therefore be interpreted with caution.

CSF analysis via PCR also demonstrated a fair sensitivity of 71% in our cohort, while PAS staining of the CSF showed much lower diagnostic yield, with only one-third of cases testing positive. These findings are consistent with the literature, which attributes the low sensitivity to the scarcity of *Tropheryma whipplei* in CSF, making the confirmation of neurological involvement particularly challenging [51,52,55,56] with this method.

Despite the limitations of CSF PCR, in our view, it remains the best trade-off between low invasiveness and diagnostic yield, especially when duodenal or brain biopsy is not considered as an option. To increase diagnostic accuracy, we recommend repeated non-invasive sampling from different sources, including saliva, stool, CSF, and other accessible tissues if warranted. These findings align with recent literature calling for multi-sample, multimodal diagnostic approaches, especially in patients without gastrointestinal involvement [4].

Notably, one patient initially had negative PCR results in the CSF, duodenal biopsy, and serum, despite presenting with cerebellar atrophy and ophthalmoplegia. Although also suffering from uveitis, a definitive diagnosis was achieved only after PCR testing of the vitreous humor. A second CSF sample taken at the same time also tested positive for *Tropheryma whipplei*. The diagnosis was thus confirmed eight years after symptom onset, illustrating the need for flexibility in sample selection when standard sites are negative but clinical suspicion remains high [33].

### Differential Diagnosis

A differential diagnosis of WhD is progressive supranuclear palsy (PSP), a sporadic neurodegenerative disorder with different subtypes, characterised in its most typical form (Richardson’s type) by vertical SGP, pseudobulbar palsy, parkinsonism, postural instability and cognitive issues [57]. Because CNS-WhD can mimic the clinical presentation of PSP (“pseudo-PSP” syndrome), the latest diagnostic criteria for PSP explicitly list Whipple’s disease as an exclusion criterion [58].

Subtle clinical signs in eye movement abnormalities can distinguish CNS-WhD on clinical grounds from PSP. In both WhD and PSP, vertical saccades are slowed (O2 criterion for PSP), but in PSP the range of voluntary vertical movements is characteristically limited, which is not the case in CNS-WhD, where smooth pursuit movements remain intact. Also, square wave jerks (O3 criterion for PSP) are absent in CNS-WhD [59]. Finally, vestibulo-ocular reflexes remain intact in CNS-WhD, same as in early stages of PSP ; but in PSP, at later stages, vestibulo-ocular reflexes are lost [58,59]. Of course, the presence in 25% of cases of the pathognomic oculomasticatory myorhythmia is a particularly distinctive feature of CNS-WhD and should immediately exclude PSP from differential diagnosis [38].

Another important differential diagnosis to consider, particularly due to its potential therapeutic implications, is anti-IgLON5 disease [60]. Similar to CNS-Whipple’s disease, it can present with a broad variety of MDs, including facial or abdominal dyskinesia, chorea, ataxia and parkinsonism; MDs have been reported in nearly two-thirds of patients [61]. These motor features of anti-IgLON5 disease are frequently accompanied by sleep disturbances, bulbar symptoms, and neuropsychiatric changes, all signs that can be found in CNS-WhD too. Of note, anti-IgLON5 disease, presenting with supranuclear gaze palsy and parkinsonism, is itself a common differential diagnosis for PSP (14% of cases in a series of 72) [62]. A progressive supranuclear palsy–like presentation with facioskeletal myorhythmia, but without sleep disturbance, has also been documented [63]. Additionally, myorhythmia has been reported to persist during both wakefulness and sleep in a case of anti-IgLON5 disease, an unusual finding also noted in early descriptions of CNS-WhD [38,64,65].

In conclusion, the type of MDs may orient the differential diagnosis between CNS-WhD and PSP, less conclusively between CNS-WhD and anti-IgLON5, the latter being a common differential for PSP. Hyperkinetic movements are frequent in both CNS-WhD (myoclonus, myorhythmia) and anti-IgLON5 (chorea, facial myokymia) and uncommon in PSP, which is primarily characterized by axial parkinsonism, apart from the SGP. Therefore, the presence of SGP accompanied by hyperkinetic movements should raise suspicion for CNS-WhD or anti-IgLON5 disease rather than PSP, particularly when accompanied by sleep disturbances.

However, sleep disorders again are not a reliable distinguishing factor, as they are frequently reported in all three conditions. Interestingly, sleep disturbances occurred in over 75% of patients with PSP from a series of 30 cases [66], despite not being part of the formal diagnostic criteria [58]. Although PSP is a tauopathy, not an alpha-synucleinopathy, which are commonly associated with sleep-related symptoms, especially REM-sleep behavioural disorder, this high prevalence is likely attributable to neurodegeneration and atrophic changes within the brainstem [67].

### Terminology

Terminological ambiguity surrounds several movement phenomena that can occur in CNS-WhD, and may lead to under recognition and under reporting. One important source of terminological ambiguity is the distinction between myokymia and myorhythmia, which are sometimes used interchangeably in the literature, contributing to diagnostic confusion:

- Myokymia classically denotes continuous, vermicular (“wormlike”) muscle fiber rippling visible under the skin. It consists of irregular, undulating contractions (often in facial or limb muscles) driven by peripheral nerve hyperexcitability, with electromyography (EMG) showing characteristic grouped motor unit discharges (myokymic bursts) at irregular intervals [68].
- In contrast, myorhythmia is a type of rhythmic myoclonus and describes a repetitive, rhythmic, slow oscillatory movement (~1–4 Hz) reflecting its origin in central oscillator lesions (brainstem, thalamus or diencephalon) such as Whipple’s encephalopathy, stroke or encephalitis [40,41]. In the context of CNS-WhD, the term myorhythmia appears prominently in the literature, largely due to oculomasticatory myorhythmia being a well-recognized and highly suggestive clinical hallmark.

A second terminological ambiguity lies in the distinction of myoclonus and myorhythmia. Ure et al. define myorhythmia as a type of rhythmic myoclonus; and this definition is adopted here [69].

Another common debate is whether to use the wording “palatal tremor” or “palatal myoclonus”. This movement disorder phenomenon refers to involuntary rhythmic oscillation of the soft palate (often ~1–2 Hz, with variable frequency). Two subtypes are recognized:

- an essential form: absence of structural lesion; typically causing ear clicks from tensor veli palatini contractions and
- a secondary symptomatic form: caused by lesions in the dentato-rubral-olivary loop, i.e. Guillain–Mollaret triangle, leading to levator veli palatini activation and often MRI-detectable inferior olivary hypertrophy) [70].

The term “tremor” has been retained in some consensus to emphasize its continuous, oscillatory nature. However, unlike classic tremors, palatal tremor does not oscillate around a fixed midpoint, which blurs the distinction between tremor and segmental myoclonus.

Biller & Espay, in their nosography of the “essential” – volitional palatal tremor [71], use the term myorhythmia for the lesional secondary palatal tremor. The use of this terminology is considered appropriate, as this type of MDs truly represents a type of “rhythmic myoclonus”. For this reason, myorhythmia was included under the broader umbrella of myoclonus in the present study.” Interestingly, a patient with confirmed CNS-WhD was reported as suffering from both perioral myokymia and palatal tremor, the latter being considered under the umbrella “rhythmic myoclonus” (see above) [72], but the former not. Myokymia, although a true peripheral nerve system-induced movement disorder, may in some instances be caused by central lesions downstream from the motor brainstem nucleus, which probably is the case here [73].

### Treatment and Outcome

The optimal treatment strategy for CNS-WhD remains debated, primarily due to the rarity of the condition, limiting randomized trials. Historically, the standard regimen included an initial course of intravenous ceftriaxone followed by prolonged oral trimethoprim-sulfamethoxazole (TS) [74]. However, clinical experience and case series have raised concerns about the efficacy of TS monotherapy, particularly in CNS involvement, with documented relapses and treatment failures [75], probably due to inadequate CNS penetration [29,76]. In response to these limitations, alternative regimens emerged, notably the use of doxycycline in combination with hydroxychloroquine, followed by lifelong doxycycline prophylaxis, aiming to enhance intracellular bactericidal activity. Reports have supported the potential efficacy of this regimen in isolated CNS-WhD cases [28]. Nevertheless, some case reports have described poor responses and continued progression under doxycycline–hydroxychloroquine therapy, prompting for more aggressive initial therapy, such as the combination of ceftriaxone and streptomycin [10].

In our cohort, a combination of TS and ceftriaxone was primarily used in approximately two-thirds of patients. However, our review shows increasing adoption of doxycycline combined with hydroxychloroquine, with a notable increase in its use since the last update in 2018. This shift aligns with findings of the recently published phase 2/3 randomized, open-label, non-inferiority trial conducted by Moos et al. [30]. This trial directly compared intravenous ceftriaxone followed by 12 months of oral TS with a fully oral regimen of doxycycline and hydroxychloroquine for 12 months. The study found no significant difference in clinical outcomes, suggesting that the oral regimen may be a viable alternative to traditional therapy. Importantly, the study included a subset of patients with CNS involvement, allowing for evaluation of neurological outcomes.

To the best of our knowledge, no study has directly compared the efficacy of initial intravenous ceftriaxone followed by long-term doxycycline combined with hydroxychloroquine, despite this regimen being recommended as an alternative, particularly in cases of intolerance to TS [4].

Ultimately, the treatment of CNS-WhD continues to be guided more by expert opinion than by robust clinical trial data. An important practical point is that periodic PCR re-testing (e.g., CSF or other involved tissues) is recommended to monitor and adapt treatment, and to guide the cessation of therapy to reduce the risk of relapse. Further reports including CNS-involved cases with longer follow-up are essential to clarify the most effective treatment strategies.

Treatment outcomes varied, with 53% of patients showing improvement in MDs. However, inconsistencies in the reporting of detailed outcome measures limited the ability to draw firm conclusions about responses to individual symptoms. Our findings support previous observations that early initiation of antibiotic therapy is associated with better neurological recovery [33,77].

### Implications and Recommendations for Clinical Practice

This review highlights critical features that should raise suspicion for CNS-WhD, particularly when the following clinical tetrad is present: progressive MDs (especially OMM/OFSM, ataxia or other hyperkinetic movements encompassed in the broad family of myoclonus), vertical SGP, cognitive or neuropsychiatric changes and sleep disturbances, even in the absence of systemic findings.

Concerning diagnosis, we recommend that clinicians maintain a low threshold for ordering CSF PCR as a first-line investigation, particularly in the presence of neurological symptoms suggestive of CNS-WhD. If initial results are negative, repeated non-invasive sampling of saliva or stool for PCR should be pursued. When suspicion remains high, PAS staining and PCR of duodenal biopsy specimens should be considered as the next step. Brain tissue biopsy, while regarded as the diagnostic gold standard, should be reserved for cases where less invasive methods are inconclusive, and the diagnostic benefit clearly outweighs procedural risk. Early identification and appropriate antimicrobial therapy remain the best strategy to mitigate morbidity and mortality.

## Limitations

This study has several limitations inherent to case-based systematic reviews. First, data heterogeneity and inconsistent reporting limited analytical approaches. Only 130 of the 168 cases included sufficiently detailed clinical descriptions for inclusion in quantitative analyses. Second, the reliance on published reports introduces publication bias, as milder or non-pathognomonic cases may be underreported. Finally, outcome measures were inconsistently described, preventing nuanced analysis of treatment efficacy.

## Conclusion

This systematic review highlights the central role of MDs and oculomotor abnormalities in the diagnosis of CNS-WhD. SGP alongside myoclonus (including the rhythmic myoclonus “myorhythmia” and its famous subtypes OMM and OFSM), emerged as the most frequent and diagnostically significant features. These signs, when recognized early, can prompt timely diagnostic workup and targeted treatment, even in the absence of systemic symptoms. These findings reinforce the importance of detailed neurological examination and raise awareness of these underrecognized, yet indicative signs. Greater standardization in reporting is needed to refine diagnosis and improve outcomes in this rare but treatable condition.

## Additional File

The additional file for this article can be found as follows:

10.5334/tohm.1075.s1 Supplementary Material. Supplemental methods and table 1.

## Abbreviations

CNS = Central nervous system

CSF = Cerebrospinal fluid

MRI = Magnetic resonance imaging

MDs = Movement disorders

OFSM = Oculofacioskeletal myorhythmia

OMM = Oculomasticatory myorhythmia

PAS = Periodic acid-Schiff

PCR = Polymerase chain reaction

PEO = Population, Exposure, and Outcome

PSP = Progressive supranuclear palsy

SGP = Supranuclear gaze palsy

TS = Trimethoprim-Sulfamethoxazole

WhD = Whipple’s disease
