# Supplementary material for: Movement Disorders and Oculomotor Abnormalities in Whipple’s Disease: An Updated Systematic Review
Source: Tremor Other Hyperkinet Mov (N Y). 2025 Nov 11;15:57. doi: 10.5334/tohm.1075 (PMC12617415; doi:10.5334/tohm.1075)
Supplement: Supplementary Material. — Supplemental methods and table 1. [file tohm-15-1-1075-s1.pdf]

# SUPPLEMENTAL MATERIAL

## Movement disorders and oculomotor abnormalities in Whipple's disease: an updated systematic review

Errikos Maslias[1]* Ruben Anker[2]*, Philip Euskirchen[3,4], Karin Diserens[1], Julien F. Bally[1]

1. Service of Neurology, Department of Clinical Neurosciences, Lausanne University Hospital (CHUV) and University of Lausanne, Lausanne, Switzerland
2. University of Lausanne, Lausanne, Switzerland
3. German Cancer Consortium (DKTK), Partner Site Berlin, German Cancer Research Center (DKFZ), Heidelberg, Germany
4. Department of Neuropathology, Charité-Universitätsmedizin Berlin, corporate member of Freie Universität Berlin and Humboldt Universität zu Berlin, Berlin, Germany

*First Co-authors, contributed equally*

## Supplemental methods

**Definitions of Clinical Signs and Symptoms** (31)

*Oculomasticatory myorhythmia (OMM)* is characterized by smooth, continuous, slow (1-3 Hz), pendular, convergent-divergent ocular movements and concurrent contractions of the masticatory muscles.

*Oculofacioskeletal myorhythmia (OFSM) are i*dentical, but also involve rhythmic movements of the proximal and distal skeletal muscles. Despite this difference, the terms OMM and OFSM are sometimes used interchangeably.

For the purpose of this systematic review, clinical descriptions of similar phenomena were still classified under these categories, even if the authors did not use the exact terms. Both OMM and OFSM are considered pathognomonic for Whipple’s disease.

The *myoclonus* category also encompasses related terms, such as jerks, while *appendicular ataxia* includes description of uncoordinated limb movements.

*Upper motor neuron syndrome*, includes all first motor neuron signs, such as Babinski sign, hyperreflexia, pyramidal weakness, and spasticity.

Increased appetite and weight gain were grouped under "*hypothalamic dysfunction*," but sleep disturbances were listed separately.

*Neuropsychiatric symptoms* include any neuropsychiatric alterations, such as depression, personality changes and apathy.

Finally, the term *encephalopathy* was used to describe acute or subacute syndromes, in contrast to *cognitive problems* which referred to chronic or progressively worsening conditions.

**Supplemental table 1 : Non-MD clinical signs and symptoms in CNS-WhD patients suffering from at least one movement disorder (MD) or supranuclear gaze palsy**

| | Single cases (n 130) | % | Case series (n = 38) | % | Total (n =168) |
|---|---|---|---|---|---|
| **CNS-WhD with systemic signs and symptoms \| isolated CNS-WhD** | 105 \| 25 | 81 \|19 | NA | NA | - |
| **NON-MD NEUROLOGICAL FEATURES** | | | | | |
| Cognitive problems (any cognitive domain, isolated or not) | 90 | 69 | 29 | 73 | 119 |
| Encephalopathy (includes both mental status changes and vigilance changes: disorientation, confusion or delirium, diminished level of consciousness or drowsiness, lethargy, stupor, coma…) | 67 | 52 | 3 | 8 | 70 |
| Upper motoneuron syndrome | 40 | 31 | 13 | 34 | 53 |
| Dysarthria (type often not specified) | 38 | 29 | 4 | 11 | 42 |
| Neuro-psychiatric symptoms (including hallucinations) | 54 | 42 | 3 | 8 | 57 |
| Sleep disorders (including insomnia, hypersomnia, sleep apnea syndrome and sleepiness) | 52 | 40 | 7 | 18 | 61 |
| Seizures | 20 | 15 | 5 | 13 | 25 |
| Headache | 21 | 16 | 0 | 0 | 21 |
| **Miscellaneous** (< 10% occurrence) | | | | | |
| 1. sensory impairment | 8 | 6 | 0 | 0 | - |
| 2. ptosis | 11 | 8 | 0 | 0 | - |
| 3. visual acuity problem | 9 | 7 | 0 | 0 | - |
| 4. dysphagia | 7 | 5 | 0 | 0 | - |
| 5. facial palsy/paresis without precision or precision that it is peripheral in origin | 2 | 2 | 0 | 0 | - |
| 6. vertigo | 4 | 3 | 1 | 3 | 5 |
| 7. hearing loss | 2 | 2 | 0 | 0 | - |

| | | | | | |
|---|---|---|---|---|---|
| 8. Soft palate paresis | 2 | 2 | 0 | 0 | - |
| 9. neuropathy | 3 | 2 | 0 | 0 | - |
| 10. amyotrophy | 1 | 1 | 0 | 0 | - |
| 11. bulbar palsies | 1 | 1 | 0 | 0 | - |
| 12. Klüver-Bucy syndrome | 1 | 1 | 0 | 0 | - |
| 13. trigeminal neuralgia | 1 | 1 | 0 | 0 | - |
| 14. bilateral wrist-drop | 1 | 1 | 0 | 0 | - |
| 15. Non specified muscular weakness | 7 | 6 | 0 | 0 | - |
| 16. urinary incontinence | 2 | 2 | 0 | 0 | - |
| 17. blurred vision | 11 | 8 | 0 | 0 | - |
| **Hypothalamic dysfunction** (sleep not included) : total | 21 | 16 | 5 | 13 | 26 |
| 1. unspecified | 2 | 2 | 5 | 13 | 7 |
| 2. polydipsia | 2 | 2 | 0 | 0 | - |
| 3. impotence/libido loss | 7 | 5 | 0 | 0 | - |
| a. origin of impotence not specified | 5 | 4 | - | - | - |
| b. due to libido loss (therefore most probably hypothalamic) | 2 | 2 | - | - | - |
| 4. weight gain/hyperphagia | 8 | 6 | 0 | 0 | - |
| 5. anorexia | 2 | 2 | 0 | 0 | - |
| 6. hypothermia/hyperthermia | 2 | 2 | 0 | 0 | - |
| **SYSTEMIC SIGNS AND SYMPTOMS** | | | | | |
| Arthritis/arthralgias | 50 | 38 | 6 | 16 | 56 |
| Gastro-intestinal symptoms (including diarrhea) | 53 | 41 | 6 | 16 | 59 |
| Fever | 45 | 35 | 4 | 11 | 49 |
| Weight loss (whatever the cause) | 63 | 48 | 2 | 5 | 65 |
| Any cardiac disorder (e.g., pericarditis, valve endocarditis, murmur, peripheral oedema…) | 7 | 5 | 0 | 0 | 7 |
| Lymphadenopathy (also as demonstrated by imaging or on autopsy) | 23 | 18 | 1 | 3 | 24 |
| Hyperpigmentation of skin | 7 | 5 | 0 | 0 | 7 |
